# P-2011. Evaluating the Impact of Penicillin Allergy Cross-Reactivity Alert Removal on Antibiotic Prescribing and Utilization

**DOI:** 10.1093/ofid/ofaf695.2175

**Published:** 2026-01-11

**Authors:** Alexander Miller, Yi Guo, Kelsie Cowman, Priya Nori, Mei Li

**Affiliations:** Montefiore Medical Center/Albert Einstein College of Medicine, New York, New York; Montefiore Medical Center, Bronx, New York; Montefiore Medical Center, Bronx, New York; Montefiore Health System, Bronx, NY; Montefiore Medical Center, Bronx, New York

## Abstract

**Background:**

Patients with a documented penicillin (PCN) allergy are often prescribed second-line antibiotics due to electronic Health Records (EHRs) warnings about cross-reactivity with cephalosporins, despite the low risk. This PCN allergy-cephalosporin cross-reactivity alert can discourage beta-lactam use, leading to negative outcomes. In June 2024, the Montefiore Health System removed this alert to improve beta-lactams use. This study evaluates the impact of this removal on antibiotic prescribing and utilization.

**Figure 1:**
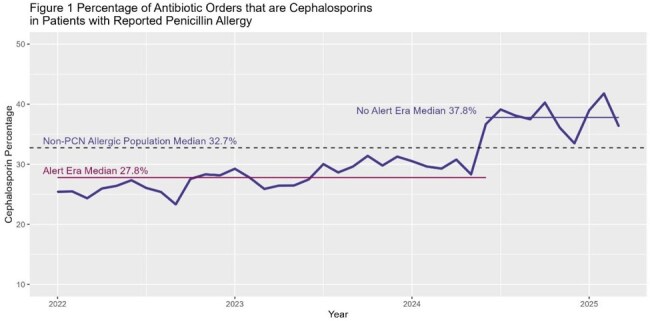
Percentage of antibiotic orders that are cephalosporins in patients with reported penicillin allergies

**Figure 2:**
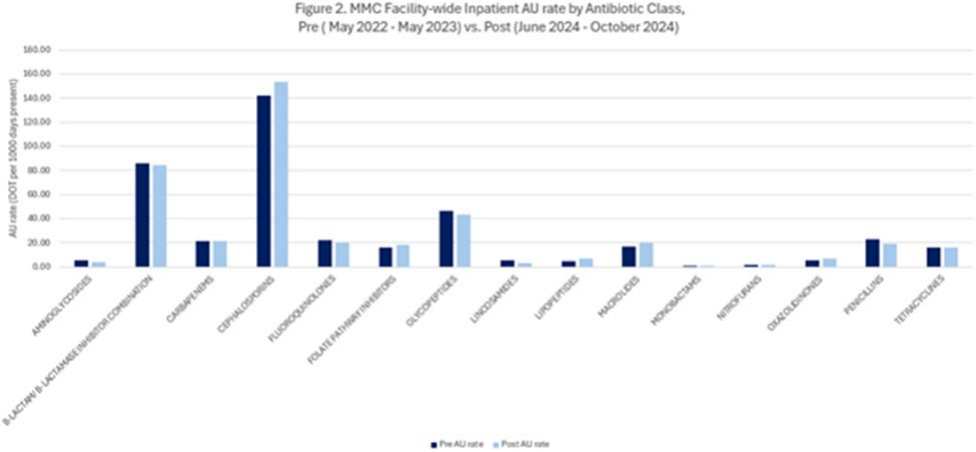
MMC Facility wide inpatient AU rate by antibiotic class

**Methods:**

This retrospective, pre-post study included patients admitted to Montefiore Medical Center who had an order for antibiotics. Antibiotic prescribing patterns and administrations were compared in patients with documented PCN allergies before (Jan-Jun 2024) and after (Jun-Apr 2025) the removal of allergy alert, and in non-PCN allergic patients as a reference group. Data collected included antibiotic class orders, antibiotic utilization rate (AU, measured as days of therapy/1000 days present), incidence of new cephalosporin allergies, and allergy severity.

**Figure 3:**
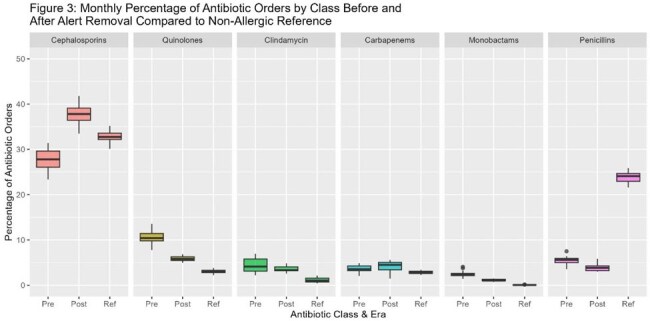
Monthly percentages of antibiotic orders by class among penicillin allergic patients before and after alert removal compared to non-penicillin allergic reference group

**Results:**

A total of 9,863 patients were included, 6,744 (68%) of who were women. The mean (SD) age was 64 (17) years. Among patients with documented PCN allergies, cephalosporin orders increased by 10% after the intervention (27.8% vs. 37.8%; p < 0.0001) (Figure 1). Cephalosporin AU rate increased from 142.1 to 156.3, p< 0.001, figure 2. During the post-intervention period, only one mild allergic reaction to cephalosporin was reported among 4,388 PCN-allergic patients who received a total of 24,643 cephalosporin doses. Orders for quinolones, clindamycin, and aztreonam declined by 4.8% (p < 0.0001),1.8% (p=0.06), and 1.3% (p < 0.0001), respectively (Figure 3).

**Conclusion:**

Removal of EHR alerts for PCN allergy-cephalosporin cross-reactivity is associated with a significant increase in cephalosporin use in PCN allergic patients without a rise in allergic reactions.

**Disclosures:**

All Authors: No reported disclosures

